# Modulating OFC Activity With tDCS Alters Regret About Human Decision-Making

**DOI:** 10.3389/fpsyg.2021.706962

**Published:** 2021-09-08

**Authors:** Ping Yu, Xinbo Lu, Yuyou Chen, Hang Ye, Lulu Zeng, Wenmin Guo

**Affiliations:** ^1^Center for Economic Behavior and Decision-Making (CEBD), Zhejiang University of Finance and Economics, Hangzhou, China; ^2^School of Economics, Zhejiang University of Finance and Economics, Hangzhou, China; ^3^School of Economics, Jiaxing University, Jiaxing, China

**Keywords:** regret, decision-making, orbitofrontal cortex, transcranial direct current stimulation, counterfactual thinking

## Abstract

Regret is a common emotion in daily life. Humans always regret their decision-making choices if the chosen outcome is bad. Neuroscientific studies suggest that the orbitofrontal cortex (OFC) influences feelings of regret. We used a transcranial direct current stimulation (tDCS) device to study the role of regret in participants’ decision-making by modulating the activity of the OFC. The two-wheel-of-fortune gamble task was used in our experimental design, and we asked the participants to rate their feelings of regret after the computer presented the obtained and unobtained outcomes. The experimental results revealed that the effect of stimulation type was significant, which indicated that the influence of the OFC in regret was modulated by tDCS. Furthermore, based on *post hoc* analyses (Bonferroni), regret was lower in those who received left anodal/right cathodal stimulation than in those who received sham stimulation, which revealed that modulating the activity of the OFC reduced the emotional intensity of regret. In addition, an inverted U-shaped curve characterized the mean ratings of regret over time.

## Introduction

Regret is a common and distressing emotion in our daily lives that has long-lasting consequences for our health and well-being (Landman, 1987; Lecci et al., 1994; Wrosch et al., 2005; Beike et al., 2009). We always regret our decision-making when the outcomes are bad. It is hard to accomplish living a life without any regrets. Kahneman and Tversky (1982) proposed the notion of regret perception, and they created a series of story scripts to reveal people’s regret experiences. They found that it seemed to be more regrettable if a bad outcome resulted from action rather than inaction. Subsequently, Kahneman and Miller (1986) proposed the action effect of regret. They suggested that for the same loss, the feeling of regret caused by action is stronger than the feeling of regret caused by inaction. However, Gilovich and Medvec (1995) argued that actions would produce more feelings of regret over a short time period, while inactions would produce more feelings of regret over a long time period, as assessed by telephone surveys and interviews. Zeelenberg and Pieters (2007) proposed regulation regret theory, which suggested that the degree of regret depends on the relationship between the action and the situation. If the action is reasonable and appropriate in the situation, then inaction will produce stronger regret; otherwise, the action will produce regret.

Research on regret theory originated from the Allais paradox. Economists have attempted to use regret aversion to explain the Allais paradox. They suggest that people’s decision-making not only maximizes revenue but also minimizes regret. Furthermore, they have established that the regret function extended the expected utility (EU) theory (Bell, 1982; Loomes and Sugden, 1982, 1987; Zeelenberg, 1999). The regret function of EU reflects the psychology of the decision-maker in the decision-making process. The decision-maker is not only concerned with the outcome of the choice but also concerned with the outcome of the alternatives. If the outcomes of the alternatives are discovered to be better than the chosen outcome, people will feel regret even if the choice had a good result (Quiggin, 1994; Zeelenberg and Beattie, 1997).

Research on regret has been further studied by psychologists. These studies were mainly designed to explain the brain basis of and neural mechanisms underlying regret. Psychologists believe that regret is based on counterfactual thinking (Roese, 1994, 1997; Zeelenberg et al., 1998; Byrne, 2002). An example of a counterfactual thought is the following: if I had left the office earlier, I would not have missed my train. Markman et al. (1993) define this phenomenon as upward counterfactual thinking. It is about how (past) reality could have been different if another option was chosen (Van Hoeck et al., 2013). Regret takes place when people compare the outcome of a choice (reality) to the better outcome of a foregone choice that eliminated the alternatives (what might have been; Coricelli et al., 2007). Regret is usually a negative emotion with a painful lesson. It comes from higher-level cognitive processing. Guttentag and Ferrell (2004) found that 7-year-old children will experience regret when they compare the current results with the possible outcomes, while 5-year-old children will not. Cognitive neuroscience has explored the brain basis of these counterfactual processes. The counterfactual process was shown to be abolished in a group of patients with orbitofrontal cortex (OFC) damage (Camille et al., 2004).

The connection between the OFC and regret has been supported by functional imaging studies. Coricelli et al. (2005) showed that participants were regretful about the obtained outcome with complete feedback but disappointed with the obtained outcome with partial feedback using two-wheel-of-fortune gamble tasks. Furthermore, they detected the medial OFC response by functional magnetic resonance imaging (fMRI) and revealed that the level of regret correlated with the obtained outcomes and the nonobtained outcomes. Similarly, Camille et al. (2010) examined how OFC and striatum responses to counterfactual outcomes were modulated by allowing the subjects to change their minds. The results supported the notion that outcomes related to the satisfaction ratings were highly sensitive to activity in the striatum and OFC and that satisfaction ratings following losses and the striatal signal were lower when the opportunity is given to change. Using the same experimental design, researchers studied the similarities and differences among regret, relief, and disappointment (Zeelenberg, 1999; Zeelenberg and Pieters, 2004), as well as the effects of action vs. inaction on regret (Kahneman and Miller, 1986; Zeelenberg et al., 2002). Giorgetta et al. (2013) investigated the neural correlates of regret and disappointment. They experimentally induced each emotion by manipulating feedback (chosen gamble vs. unchosen gamble), agency (human vs. computer choice), and outcomes (win vs. loss) in a fully randomized design. In addition, some fMRI results have proven that regret is associated not only with the OFC (Rolls, 2000; Gottfried et al., 2003) but also with the striatum (Nicolle et al., 2011; Frydman and Camerer, 2016), hippocampus, amygdala (Eichenbaum, 2004; Anderson et al., 2006), and ventromedial prefrontal cortex (Canessa et al., 2009; Levens et al., 2014).

The OFC plays an important role in the cognitive process of human reward and punishment (Rolls, 2004). The left central area of the OFC is sensitive to rewards, and the right lateral area of the OFC is sensitive to punishment (O'Doherty et al., 2001). Through fMRI studies, it has been found that increased activity of the left central OFC could be particularly responsive to the rewarded events (Diekhof et al., 2011). Furthermore, other results of the incentive working memory task suggested that the right posterior medial OFC is particularly involved in the processing of the punishing aspect of salient events and probably mediates behavior based on negative outcomes of action (Szatkowska et al., 2011).

The OFC regulates people’s emotions and decision-making behaviors through such cognitive activities (Bechara et al., 2000). Neuroimaging research conducted by Elliott et al. (2000) proved that the medial frontal middle area and the OFC were activated when humans experience emotions. Regret is a complex social emotion in human beings that emerges from counterfactual thinking (Roese, 1997), which is also modulated by the OFC (Camille et al., 2004; Coricelli et al., 2005).

Furthermore, some studies revealed that the left OFC controls emotions, while the right OFC controls cognition. Previous studies have found that the left OFC influences automatic emotion regulation (Pourtois et al., 2006; Hua et al., 2020). Banks et al. (2007) found that subjects whose left OFC, and not the right OFC, had stronger psychosocial interactions (PPI) with the amygdala experienced fewer negative emotions. Wang et al. (2016) researched the role of the right OFC in cooperative behavior, and their tDCS stimulus results revealed that the right OFC improved human cooperation since the OFC is the specific brain region associated with guilt. Nejati et al. (2018) studied the role of the right OFC in executive functions (EF) with respect to cognitive (cold) and affective/reward-related (hot) processes. Li et al. (2010) studied the critical role of the right OFC in facilitating human olfactory consciousness. Another fMRI study showed that there are medial and lateral differences in OFC with respect to regret (Coricelli et al., 2005). They suggested that regret arose from the magnitude of the difference between unselected and selected outcomes, which was correlated with enhanced activity of the lateral OFC, and that the medial OFC contributes to the experience of regret by the provision of information regarding the outcome of the alternative nonselected gamble.

Although many studies have shown relationships between regret and activity in the brain by fMRI, how the OFC influences regret is not clear. The use of transcranial direct current stimulation (tDCS) can help identify causal relationships between activity in the OFC and regret. As Fudenberg (2006) said, even though neuroimaging proves the existence of interactions between our behaviors and cognitive processes, we should caution that the targeted brain area is not causing this behavior because the behavior of interest does not involve activity in a single brain area, but involves other upstream neurons. In other words, brain imaging results about the activity of a brain area following a behavior does not indicate that they are causally related; however, brain stimulation technologies such as tDCS and transcranial magnetic stimulation (TMS) can examine these causal relationships between the behavior and its related brain region (Zheng et al., 2021).

Our paper used tDCS to explore how OFC activity interacts with regret. The study also used two-wheel-of-fortune gamble games. Although based on Coricelli et al. (2005) and Camille et al. (2010), our experimental design incorporated the following changes: first, the two-wheel-of-fortune gamble task contains a deterministic/lower-risk gain option and a higher-risk gain option; second, we first established the option at the higher-risk, then asked the subjects to change their mind or not; third, we asked the subjects to directly rate their feelings of regret using the 10-level regret scale after the obtained and unobtained outcomes were provided by the computer. Then, we conducted the experiment. According to the research of Ouellet et al. (2015) and Yang et al. (2017), the tDCS stimulation of the OFC is conducted as follows: anodal left OFC/cathodal right OFC, cathodal left OFC/anodal right OFC, and sham anodal/cathodal OFC. Based on the different functions of the left OFC and right OFC, our experiment also utilized this stimulus. The results revealed that subjects’ feelings of regret decreased after modulating the activity of the OFC.

## Materials and Methods

### Subjects

One hundred nine right-handed healthy subjects who had no history of neurological or psychiatric problems and normal or corrected-to-normal vision were recruited. Fifty-four females and 55 males came from Zhejiang University of Finance and Economics. Their mean age was 21-year-old, which ranged from 18 to 29. Most of the subjects were undergraduates, and a few were postgraduates. They were naïve to tDCS and our decision-making tasks. The experiment lasted approximately 1h, and each participant received an average payment of 60 RMB yuan (approximately equal to 9.18 United States dollars) after the experiment finished. No participants reported any adverse side effects regarding pain in the scalp or headaches during or after the experiment. The protocol was approved by the Zhejiang University of Finance and Economics ethics committee.

### Transcranial Direct Current Stimulation

tDCS is a neuromodulatory technique that delivers a weak direct electrical current (e.g., 1–2mA) to excite or inhibit the cerebral cortex (Brunoni et al., 2012). tDCS, as a form of noninvasive brain stimulation, has been applied in humans for over 20years (Priori et al., 1998) and can modulate spontaneous neuronal activity (Fritsch et al., 2010). It functions by changing the critical level of excitability through two saline-soaked surface sponge electrodes (5cm×7cm) attached to the scalp, by which anodal stimulation enhances cortical excitability, and cathodal stimulation reduces cortical excitability (Nitsche and Paulus, 2000). The application of tDCS in psychological research has mainly involved the study of specific brain areas or specific psychological problems. We used a tDCS device (NeuroConn, Ilmenau, Germany) to study the effects of cortical excitability of the OFC.

**Figure 1:**
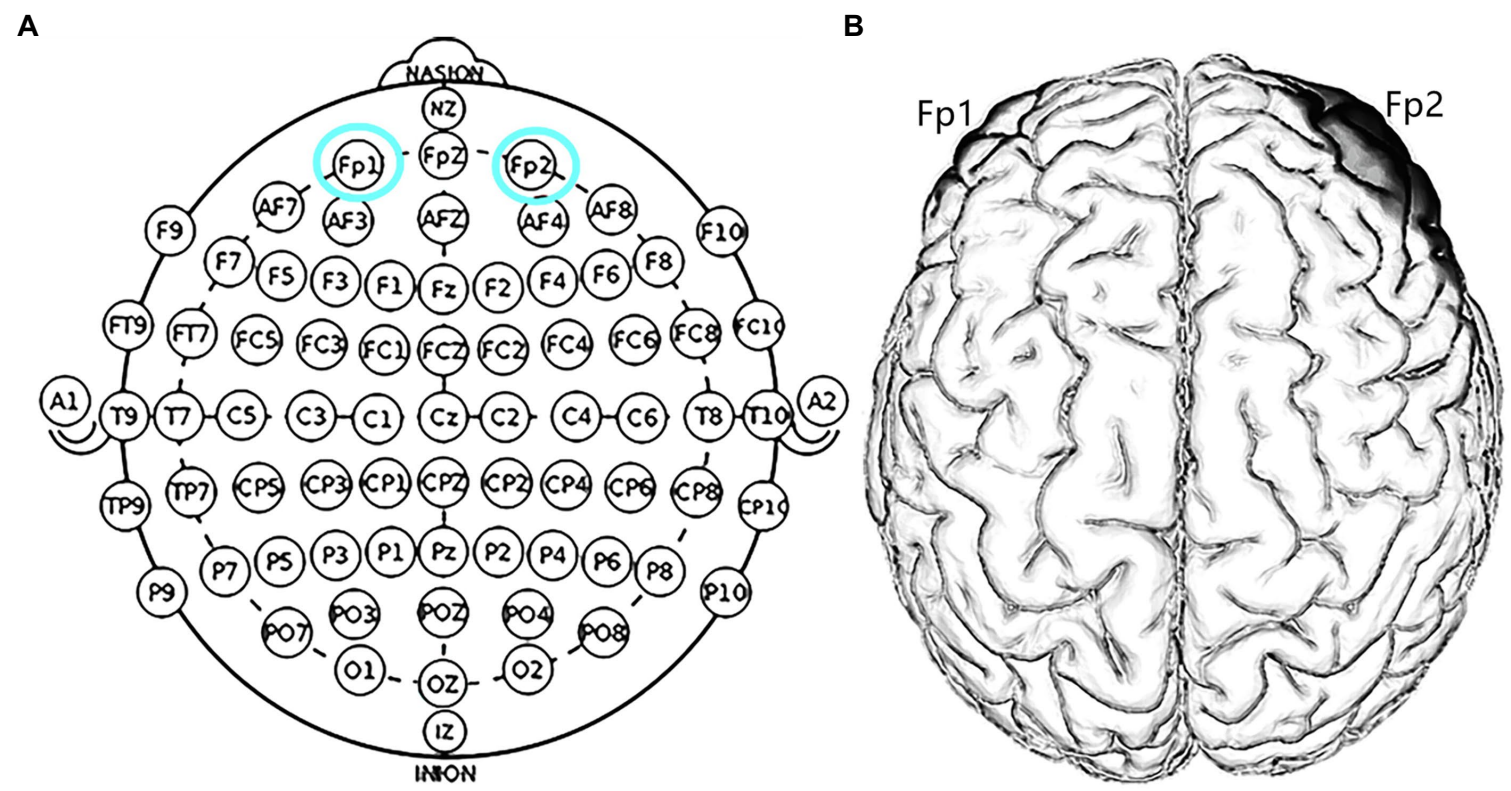
Schematic and locations of the electrode positions. **(A)** Schematic of the electrode positions Fp1 and Fp2 based on the international EEG 10–20 system. **(B)** Locations of the orbitofrontal cortex (OFC) of the human brain.

**Figure 2:**
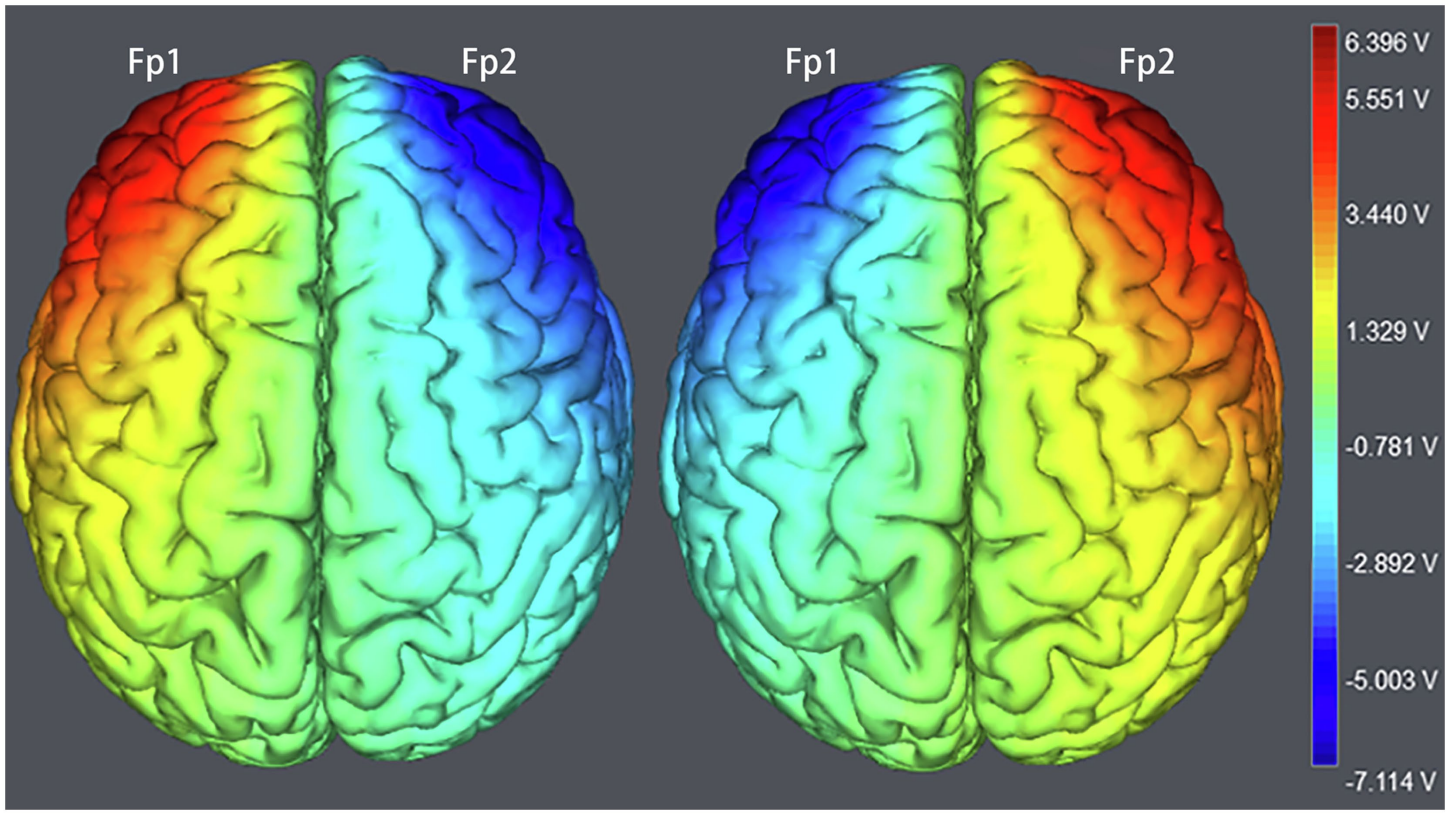
The stimulation models of transcranial direct current stimulation (tDCS) treatments. Electric field stimulation were performed with the Neuroelectrics Instrument Controller Software (version 1.3, Spain). Stimulated field intensity is indicated by the color bar. The axis represents the range of input voltage from −7.114 to 6.396v.

The target areas were localized in the right Fp2 and the left Fp1 positions based on the 10–20 EEG system (Figure 1). Regarding OFC stimulation, each participant was randomly signed to one of the three stimulation treatments: (1) the anodal electrode was placed over the right Fp2, and the cathodal electrode was placed over the left Fp1; (2) the anodal electrode was placed over the left Fp1, and the cathodal electrode was placed over the right Fp2; and (3) sham stimulation (Figure 2). Previous studies in the literature have proven that these stimulations were effective in modulating the activity of the OFC (Ouellet et al., 2015; Willis et al., 2015; Wang et al., 2016; Yang et al., 2017). The tDCS lasted 20min, with a 30-s ramp (up and down) current of 1.5mA to each OFC. For the sham stimulation, the current lasted only for the initial 30s but otherwise, the procedures were the same for the subjects. The subjects are not aware of this intensity of stimulation, and they believe that they are being stimulated by the current. This stimulation hardly modulates cortical excitability based on the short-term duration of this OFC stimulation (Gandiga et al., 2006).

### Regret Theory

Classical economic theory about human decision-making in risk choice situations is based on rational cognitive processes. According to dominant economic theory, humans’ choice among alternative options is determined by maximum EU. However, much of human decision-making (i.e., loss and risk aversion) is presumably based not only on cognitive biases but also on negative emotions (Loewenstein et al., 2001; Camerer, 2005), such as disappointment, self-blame, and regret. In recent research, an increasing number of emotional factors have been incorporated into economic and decision-making explanations. Moreover, the negative emotion of regret has been especially attractive in decision-making (regret theory; Bell, 1982; Loomes and Sugden, 1982). Regret is a negative emotion experienced when people fail to obtain better outcomes that could have been obtained if they made a different choice. Regret theory refers to incorporating regret into decision-making models to enhance EU.

Regret theory is related to decision-makers being concerned about the outcomes of their choice compared to the outcomes associated with alternatives. When they discover that choosing the alternative option would have led to better outcomes, they feel regret; otherwise, they feel rejoice. The regret function is as follows:

*x*_1_ and *x*_2_ express the outcomes of options *A*_1_ and *A*_2_, respectively. According to regret theory, the perceived value to decision-makers consists of two parts: the utility value of the current choice and the regret-rejoice value. Therefore, the perceived value of the option *A*_1_ for decision-makers is as follows:

(1) $$u\left(x_{1},x_{2}\right)=u\left(x_{1}\right)+k_{R}\times R\left(\Delta v\right)$$

(2) $$\Delta v=v\left(x_{1}\right)-v\left(x_{2}\right)$$

*k_R_* expresses the degree of the regret influence. *v*(x_1_) and *v*(x_2_) represent the utility value of the alternative option *A*_1_ and *A*_2_ by the decision-maker, respectively. *R*(Δ*v*) indicates the regret-rejoice value, which is an indicator that the decision-maker is pleased to choose option *A*_1_ and give up option *A*_2_. If *R*(Δ*v*)>0, *R*(Δ*v*) refers to the value of rejoicing; if *R*(Δ*v*)<0, *R*(Δ*v*) refers to the value of regret, which is an indicator that the decision-maker regrets choosing option *A*_1_ and not choosing option *A*_2_.

Suppose the *v*(*x*) function is as follows:

(3) $$v\left(x\right)=1-\exp\left(\beta x\right),\;0<\beta <1$$

*β* is the risk aversion coefficient, the greater the beta value is, the greater the risk aversion of the decision-maker. In the regret theory of the decision-making process, the final choice order is based on the multiattribute comprehensive perceived utility value of each option. Our following experimental design is based on regret theory.

## Experimental Design

### Gambling Task

**Figure 3:**
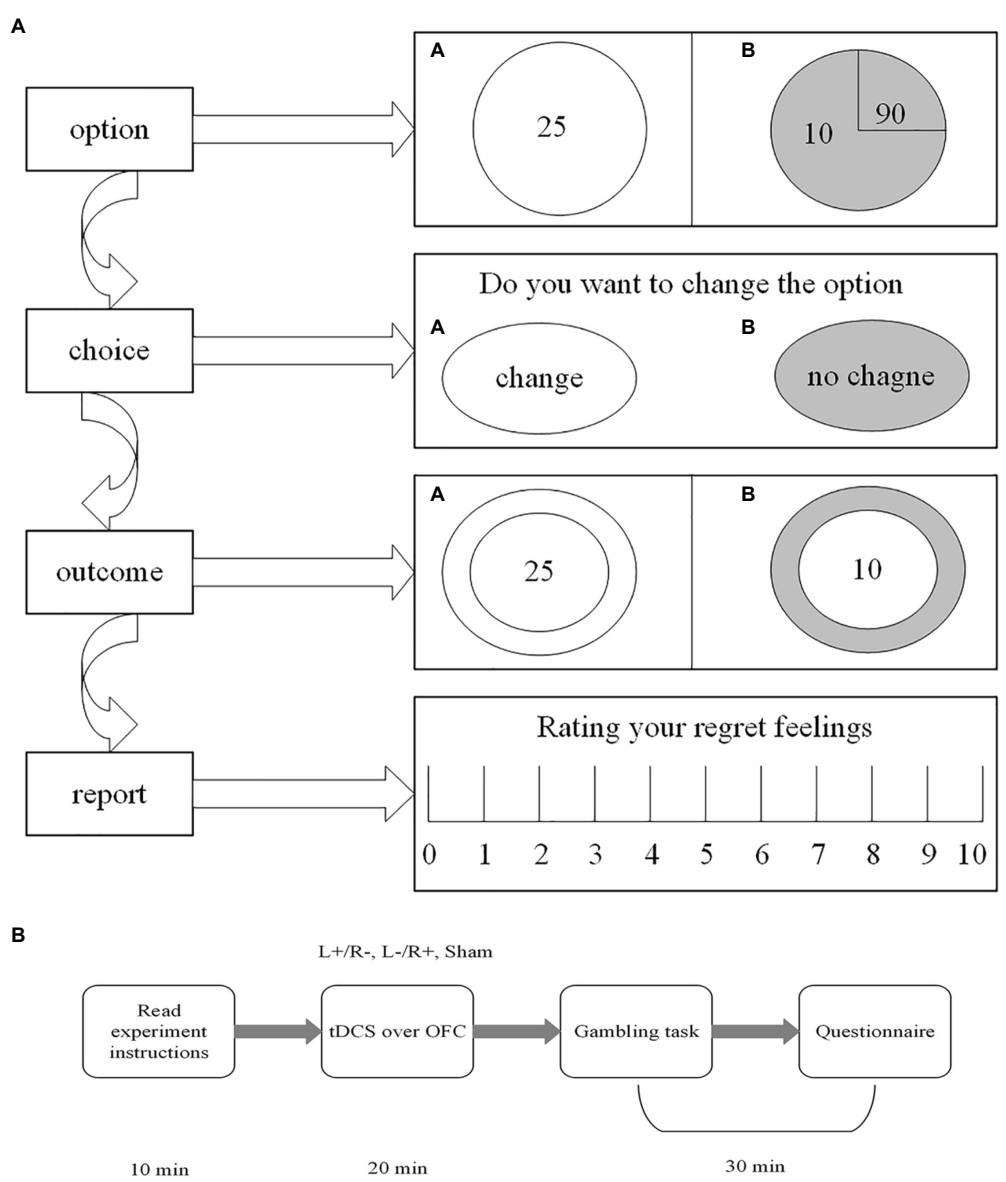
The gambling task and the experimental procedure. **(A)** The gambling task. First, option B was established by computer. Second, the subjects were asked whether they wanted to change the computer’s option. Third, the obtained outcome and the unobtained outcome were presented by the computer. Fourth, the participants were asked to report their regret feelings. **(B)** The experimental procedure.

Our gambling task is modified from Camille et al. (2004) and Coricelli et al. (2005) and involves real monetary wins and losses that are dependent on the choices made during the task. Two options appeared on the computer screen (option A and option B). Each option was composed of two factors associated with different possibilities of outcomes. Usually, option A has a determined item, with a 100% probability. Option B is a risk item with an uncertainty probability. The participants were presented with a choice between option A and option B. In addition, it needs to be pointed out that we first established option B as the chosen option and then asked the participants whether they wanted to change to the other option. They reevaluated options A and B and then selected one of the two options (change vs. no change) by clicking the mouse. Once the participants clicked the mouse and made the decision, the outcome associated with the chosen option was presented on the computer screen. As described by regret theory, regret takes place when individuals compare the obtained outcome with the unobtained outcome; therefore, the alternative option with the unobtained outcome was also presented, which allowed the subjects to view both outcomes. At the end of each gambling task, the subjects reported their regret using a rating scale from 0 (no regrets at all) to 10 (extremely regretful). Figure 3A shows the gambling task designed for our experiment to study participants’ feelings of regret.

### Procedure

The entire experiment contains three steps. In step 1, the subjects were randomly assigned to seats. Then, there was a 20-min tDCS stimulation for the participants, and each participant received one of the three stimulations (left anodal/right cathodal, left cathodal/right anodal, and sham). In step 2, after the tDCS stimulation, there were six trials of the gambling task for the participants to accomplish. Table 1 shows the six trials used in the gambling task programmed in software z-tree (Fischbacher, 2007). Unlike Camille et al. (2004) and Coricelli et al. (2005), different numbers of each trial were incorporated into the design of our gambling task. The purpose of this design was to induce decision-makers to experience more regret and to truthfully report these feelings. In step 3, after the gambling task, the subjects were given a questionnaire to complete, which collected personal information, such as gender, age, income, risk preferences, and so on. After the whole experiment, the participants received their final payments, which were determined by their choices during the experimental tasks. The payments are a combination of the show-up fee and the amount obtained in the six trials of the gambling tasks. Figure 3B illustrates the whole experimental procedure.

**Table 1:** Gamble pairs used in the experimental tasks.

Trials	Option A (lower-risk)	Option B (higher-risk)
1	100% win 35	65% win 20; 35% win 80
2	100% win 30	70% win 15; 30% win 85
3	50% win 15; 50% loss 5	50% win 20; 50% loss 10
4	100% win 20	80% win 5; 20% win 95
5	100% win 40	60% win 25; 40% win 75
6	50% win 10; 50% loss 2	50% win 14; 50% loss 6

### Data Analysis

First, we performed regression analyses to assess the effects of stimulation (left anodal/right cathodal, left cathodal/right anodal, sham) and option (change and no change). These analyses predicated the regret degree of each participant *i* with the following equation.

$$y=\beta_{0}+\beta_{1}*D_{1i}+\beta_{2}*D_{2i}+\beta_{3}*X_{j}+\varepsilon_{i}$$

*D*_1i_ are dummy-coded variables those are set to 1 if participant *i* received left anodal/right cathodal or left cathodal/right anodal stimulation, and the parameters *β*_1_ quantify the change in the regret degree due to left anodal/right cathodal and left cathodal/right anodal stimulation relative to the sham group. *D*_2*i*_ is a dummy-coded variable that is set to 1 if participant *i* changed the option, and the parameters *β*_2_ quantify the change in the regret degree due to the option. Furthermore, personal characteristics, such as gender, age, income and consumption, were also included in these analyses to capture the effects of personal characteristics.

Second, if the impact of stimulation was robust *via* regression analyses, we conducted the Kruskal-Wallis test to assess the causal relationship between the regret degree and OFC activity. If a significant difference appeared in the regret degree among the three stimulation conditions, *post hoc* analyses (Bonferroni) were performed to identify specific differences.

Finally, we performed data analysis regarding the choice and the mean values of the regret ratings across three stimulation types to assess whether there was a consistent tendency from trial 1 to trial 6.

SPSS software was used to statistically evaluate all data (version 26, SPSS Inc., Chicago, IL, United States). The significance level for all analyses was set at 0.05. Means (M) and SE of the data for the regret degrees under three conditions are shown in Table 2.

**Table 2:** Mean (M) and SE of regret dataset under three conditions.

Stimulation		Trial 1	Trial 2	Trial 3	Trial 4	Trial 5	Trial 6
L−/R+	M	3.32	4.08	4.81	4.00	3.24	2.87
	SE	0.37	0.41	0.45	0.47	0.42	0.49
L+/R−	M	2.44	3.03	3.03	3.64	2.03	1.97
	SE	0.34	0.38	0.46	0.49	0.41	0.44
Sham	M	4.67	5.97	6.08	5.72	4.06	3.61
	SE	0.42	0.49	0.48	0.52	0.47	0.52

## Results

### tDCS Results: Stimulation Effect

First, we regressed the regret degree using stimulation type and option as independent variables for each trial individually. The regression results are shown in columns 1–3 of Tables 3 and 4. We found that left anodal/right cathodal stimulation significantly decreased the regret degree compared with that in the sham group in trials 1–6; left cathodal/right anodal stimulation significantly decreased the regret degree relative to the sham group in trials 1, 2, and 4; and a significant effect of the option was found in trial 5. Furthermore, we also assessed the effects of the personal characteristics, and the results are shown in columns 4–6 of Tables 3 and 4. No significant effect of the personal characteristics was observed in any trial. Significant effects of stimulation in these models were consistent with those in the above models. In summary, these results indicated that the impact of stimulation was significant and robust.

**Table 3:** The coefficients and significances of the regression models (trials 1–3).

Regressor	Base group: sham coeff. (*p*)					
	Trial 1	Trial 2	Trial 3	Trial 1	Trial 2	Trial 3
L+/R−	−0.420^***^	−0.480^***^	−0.477^***^	−0.415^***^	−0.479^***^	−0.484^***^
L−/R+	−0.249^*^	−0.310^**^	−0.198	−0.240^*^	−0.308^**^	−0.209
Sham	---	---	---	---	---	---
Change	0.150	0.045	−0.009	0.151	0.055	0.004
Gender	---	---	---	0.084	0.018	−0.018
Age	---	---	---	−0.059	0.035	0.070
AFI	---	---	---	−0.104	−0.023	−0.072
Consumption	---	---	---	0.003	0.059	0.071
Constant	4.302^***^	5.808^***^	6.104^***^	6.306^*^	4.286	3.674

AFI, annual family income.

*** *p*<0.001;

** *p*<0.01;

* *p*<0.05.

**Table 4:** The coefficients and significances of the regression models (trials 4–6).

Regressor	Base group: sham coeff. (*p*)					
	Trial 4	Trial 5	Trial 6	Trial 4	Trial 5	Trial 6
L+/R−	−0.319^**^	−0.291^**^	−0.257^**^	−0.330^**^	−0.290^**^	−0.246^*^
L−/R+	−0.268^*^	−0.078	−0.110	−0.286^*^	−0.079	−0.089
Sham	---	---	---	---	---	---
Change	−0.059	−0.231^*^	−0.152	−0.067	−0.229^*^	−0.149
Gender	---	---	---	−0.037	0.074	0.088
Age	---	---	---	0.071	0.008	−0.033
AFI	---	---	---	−0.026	0.042	−0.041
Consumption	---	---	---	−0.016	−0.028	0.099
Constant	5.885^***^	4.589^***^	4.309^***^	3.919	4.058	4.592

AFI, annual family income.

*** *p*<0.001;

** *p*<0.01;

* *p*<0.05.

Then, the Shapiro–Wilk test showed that the regret degree across the three different stimulation conditions in all trials was not normally distributed (trial 1: *p*<0.001, trial 2: *p*=0.004, trial 3: *p*<0.001, trial 4: *p*<0.001, trial 5: *p*<0.001, trial 6: *p*<0.001). Therefore, the Wilcoxon test was used to test the stimulation effect. The results of each trial are shown below individually.

In trial 1, the Kruskal-Wallis test revealed that the regret degree differed significantly between the three stimulation conditions (*χ*^2^*_d.f.2_*=15.062, *p*<0.001). *Post hoc* analyses (Bonferroni) showed that the regret degree of the left anodal/right cathodal stimulation (mean=2.444) was significantly lower than that of the sham stimulation (mean=4.667, *p*<0.001). Although the regret degree of the left cathodal/right anodal stimulation (mean=3.324) was lower than that of the sham stimulation, the difference was not significant (*p*=0.079). Moreover, there was no significant difference in the regret degree between the left anodal/right cathodal stimulation and left cathodal/right anodal stimulation (*p*=0.284).

In trial 2, the Kruskal-Wallis test revealed that the regret degree differed significantly between the three stimulation conditions (*χ*^2^*_d.f.2_*=18.431, *p*<0.001). *Post hoc* analyses (Bonferroni) showed that the regret degree of the left anodal/right cathodal stimulation (mean=3.028) was significantly lower than that of the sham stimulation (mean=5.972, *p*<0.001). Similarly, the regret degree of the left cathodal/right anodal stimulation (mean=4.081) is also significantly lower than that of the sham stimulation (*p*=0.025). However, the difference between the regret degree of the left anodal/right cathodal stimulation and left cathodal/right anodal stimulation was not significant (*p*=0.299).

In trial 3, the Kruskal-Wallis test revealed that the regret degree differed significantly between the three stimulation conditions (*χ*^2^*_d.f.2_*=18.900, *p*<0.001). *Post hoc* analyses (Bonferroni) showed that the regret degree of the left anodal/right cathodal stimulation (mean=3.028) was significantly lower than that of the sham stimulation (mean=6.083, *p*<0.001). In contrast to trial 1 and trial 2, the regret degree of the left anodal/right cathodal stimulation was lower than the regret degree following left cathodal/right anodal stimulation (mean=4.811, *p*=0.034). There was no significant difference between regret degree following left cathodal/right anodal and regret degree following sham stimulation (*p*=0.204).

In trial 4, the Kruskal-Wallis test revealed that the regret degree differed significantly between the three stimulation conditions (*χ*^2^*_d.f.2_*=9.567, *p*=0.008). *Post hoc* analyses (Bonferroni) showed that the regret degree of the left anodal/right cathodal stimulation (mean=3.639) was significantly lower than that of the sham stimulation (mean=5.722, *p*=0.011). Although the regret degree of the left cathodal/right anodal stimulation (mean=4.000) is lower than that of the sham stimulation, the difference was not significant (*p*=0.051). Moreover, there was no significant difference in the regret degree between the left anodal/right cathodal stimulation and left cathodal/right anodal stimulation (*p*=1.000).

In trial 5, the Kruskal-Wallis test revealed that the regret degree differed significantly between the three stimulation conditions (*χ*^2^*_d.f.2_*=10.211, *p*=0.006). *Post hoc* analyses (Bonferroni) showed that the regret degree of the left anodal/right cathodal stimulation (mean=2.028) was significantly lower than that of the sham stimulation (mean=4.056, *p*=0.004). Although the regret degree of the left cathodal/right anodal stimulation (mean=3.243) was lower than that of the sham stimulation, the difference was not significant (*p*=0.526). Moreover, there was no significant difference in the regret degree between the left anodal/right cathodal stimulation and left cathodal/right anodal stimulation (*p*=0.193).

**Figure 4:**
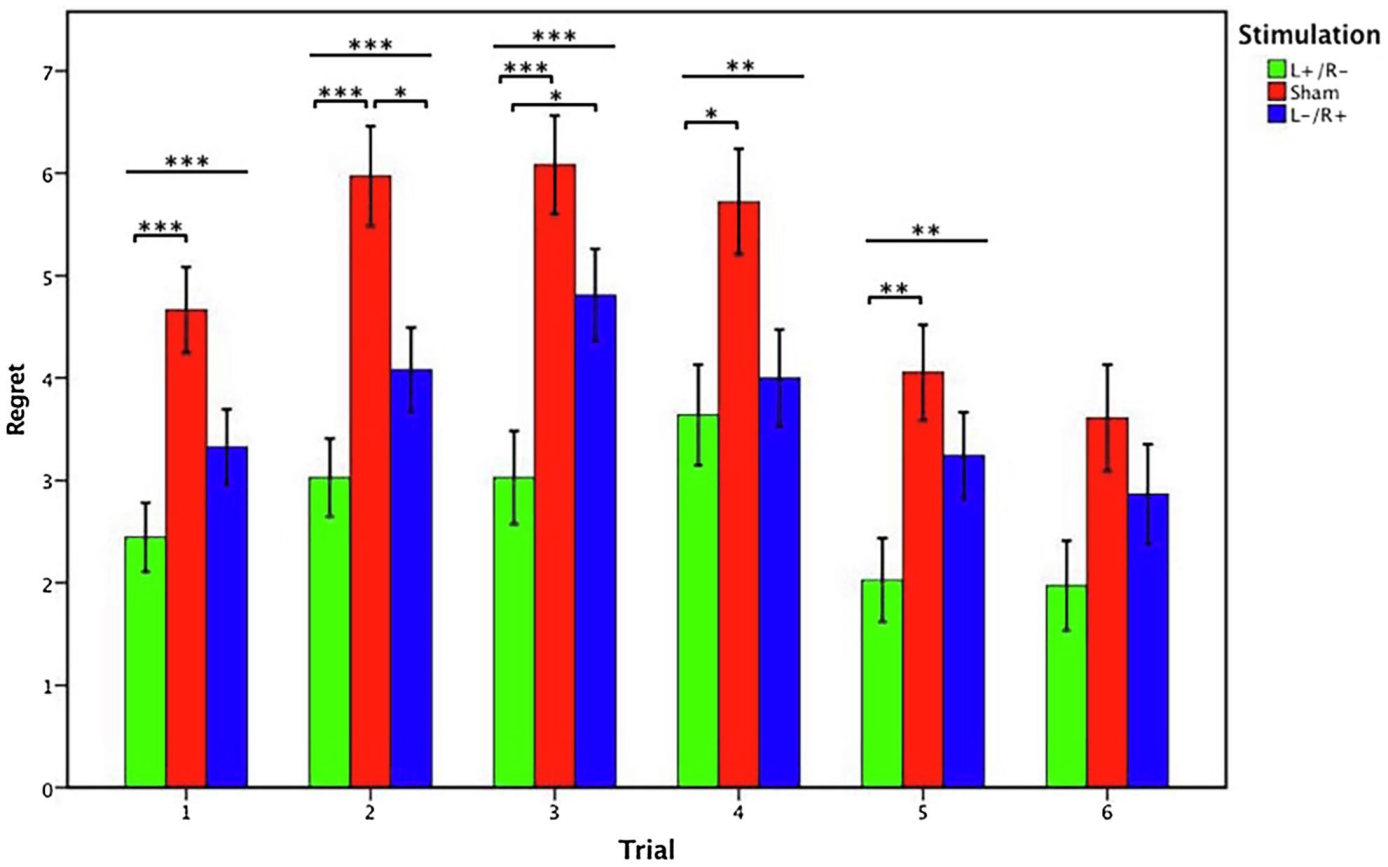
Data of regret ratings in the gambling tasks. The mean regret ratings across three stimulations from trial 1 to trial 6. Error bars represent standard error. Asterisks indicates statistically significant difference between the stimulation types.

In trial 6, no significant effect of stimulation was found in the regret degree among the three stimulation conditions (Kruskal-Wallis test, *χ*^2^*_d.f.2_*=5.872, *p*=0.053). Figure 4 presents the statistical analysis results from trials 1–6.

Overall, these results revealed that there was a significant effect of stimulation type. The regret degree associated with the left anodal/right cathodal stimulation was significantly lower than the regret degree in the sham group. The regret degree related to the left cathodal/right anodal stimulation was also lower than that in the sham group, whereas a significant difference between them was found only in trial 2. Based on these results, we confirmed that participants’ feelings of regret decreased when they received the left anodal/right cathodal tDCS stimulation. In other words, tDCS stimulation altered regret emotions in the context of human decision-making by modulating the OFC activity of the human brain.

### Data Analysis of the Relationship Between Regret and Choice

In our experiment, the variables that affected participants’ risk decision-making mainly included tDCS stimulation over the OFC, the choice about whether or not to change options. tDCS stimulation involved left anodal/right cathodal, left cathodal/right anodal, and sham conditions. Therefore, this paper provides a statistical analysis of chosen option data.

Table 5 illustrates the statistics regarding the chosen option dataset. We calculated the choice number and frequency of the options across the different types of tDCS conditions. The frequency of option B (no change) from trial 1 to trial 4 was generally higher than that from trial 5 and trial 6 under the left cathodal/right anodal and left anodal/right cathodal stimulations. Otherwise, the frequency of option A (change) from trial 1 to trial 4 was generally lower than that from trial 5 and trial 6 under the left cathodal/right anodal stimulation and left anodal/right cathodal stimulation. Furthermore, the frequency of option B (no change) under the sham stimulation in trial 1 was higher than that in trial 2, in trial 3 was higher than that in trial 4, and in trial 5 was higher than that in trial 6. However, the frequency of option A (change) under the sham stimulation yielded the opposite result to that of the frequency of option B (no change).

**Table 5:** Number (N) and frequency (F) of chosen option dataset under three conditions.

Stimulation	Option		Trial 1	Trial 2	Trial 3	Trial 4	Trial 5	Trial 6
L−/R+	B	N	22	19	16	22	11	9
		F	0.59	0.51	0.43	0.59	0.30	0.24
	A	N	15	18	21	15	26	28
		F	0.41	0.49	0.57	0.41	0.70	0.76
L+/R−	B	N	21	21	24	20	11	10
		F	0.58	0.58	0.67	0.56	0.31	0.28
	A	N	15	15	12	16	25	26
		F	0.42	0.42	0.33	0.44	0.69	0.72
Sham	B	N	18	13	23	20	21	11
		F	0.50	0.36	0.64	0.56	0.58	0.31
	A	N	18	23	13	16	15	25
		F	0.50	0.64	0.36	0.44	0.42	0.69

### Analysis of the Mean Values of the Regret Ratings

**Figure 5:**
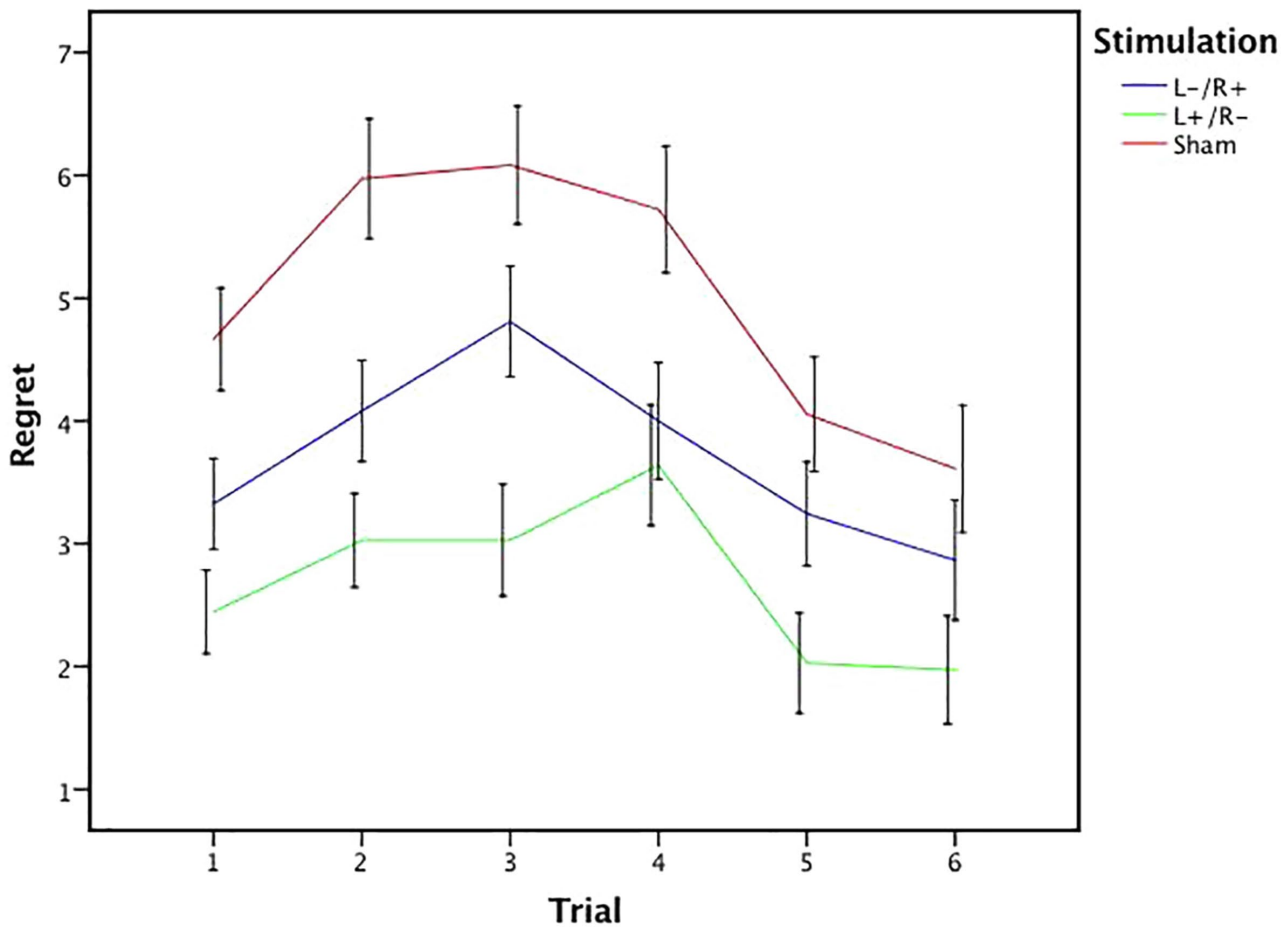
Mean value of regret ratings under different stimulation conditions. The mean regret ratings across three stimulations from trial 1 to trial 6. Error bars represent SE.

We calculated the mean rating of regret in different stimulation conditions in each trial of the gambling task for the 109 participants. Figure 5 illustrates six regret values that express the mean regret rating for the six trials of the experimental task in the three tDCS conditions. As shown in Figure 5, the sham regret value was the highest among all the datasets, the left cathodal/right anodal stimulation value followed, and the left anodal/right cathodal stimulation value was the lowest. Based on pairwise comparisons, the left anodal/right cathodal and left cathodal/right anodal values were both below the sham value, while the left anodal/right cathodal value was also below the left cathodal/right anodal value. Moreover, as the number of experimental trials increased, the degree of regret initially increased and then decreases. In other words, there was an inverted U-shaped relationship between the subjects’ regret perception and our experimental trials. Overall, we conclude that there is an inverted U relationship between the mean regret ratings and the experimental trials, regardless of the stimulation conditions leading to the obtained regret rating.

## Discussion

### Stimulation Effect

Based on the regression results, we found that there was a significant effect of stimulation type, which indicated that the stimulation conditions for the tDCS had an impact on subjects’ feelings of regret. However, there was no significant effect of the choice between the change and no change options. From the *post hoc* analyses (Bonferroni), we found that the regret degree of the left anodal/right cathodal stimulation was significantly lower than the regret degree of the sham group and the regret degree of the left cathodal/right anodal stimulation group was also lower than that of the sham group, whereas a significant difference between them was found only in trial 2.

Perhaps the research conducted by Chandrasekhar et al. (2008) and Nicolle et al. (2011) can provide an explanation for our findings. Chandrasekhar et al. (2008) noted that the medial OFC is not activated when the negative outcome is realized by participants. Nicolle et al. (2011) revealed that counterfactual thinking had no significant effect on the OFC from an fMRI study. They both argued that if subjects were sure in advance that the counterfactual thinking involved a failed event that turned out to be a negative outcome, they would experience little to no regret.

The previous study showed that anodal stimulation increases cortical excitability, whereas cathodal stimulation has the reverse effect (Utz et al., 2010). Thus, we can conclude that the left anodal/right cathodal stimulation increases the activation of the left OFC while decreasing the activation of the right OFC. Therefore, our experimental results reveal that there is a causal relationship between the left OFC and regret, which further confirmed the recent studies indicating that emotion is modulated by the left OFC (Pourtois et al., 2006; Banks et al., 2007; Hua et al., 2020).

Furthermore, based on our experimental design, there is more reward than punishment in the patient decision-making process. Our gambling task is mostly related to an obtained outcome, and even in the loss trial, each choice of obtained outcome is better than the unobtained outcome (Table 1). According to the previous studies, the left OFC is sensitive to rewards, and the right OFC is sensitive to punishment (O'Doherty et al., 2001; Diekhof et al., 2011; Szatkowska et al., 2011). Thus, our experimental design introduces more sensitivity of rewards, which in turn, under the counterfactual thinking related to a known negative outcome by the subjects, modulating the activity of the left OFC will generate less regret. However, the role of the right OFC in regret in our experiment has not yet been confirmed.

Overall, in this study, we found that the left OFC is sensitive to emotion and rewards related to regret, while the role of the right OFC in regret in our experiment has not yet been confirmed. Compared with previous studies, our research is a bilateral-stimulation neurology experiment, and we conducted three tDCS stimulation protocols (anodal left OFC/cathodal right OFC, cathodal left OFC/anodal right OFC, and sham anodal/cathodal OFC), which is more comprehensive than the protocol used by Van’t Wout and Silverman (2017). Furthermore, our results are also more definitive. According to our tDCS experiment, there is a causal relationship between regret and the left OFC, based on the emotion and rewards activated by the left OFC.

### Inverted U-Shaped Curve

Based on the mean regret rating analysis, we found that there was an inverted U-shaped relationship between regret and experimental trials. The mean value of the regret ratings initially increased and then decreased as the number of trials increased with left cathodal/right anodal and left anodal/right cathodal stimulation. Combined with the data analysis regarding choice, we find that both factors influence regret from a time perspective. The choice of change or no change has an impact on subjects’ regret perception as the continuation of experimental tasks. As the choice of task changed, the value of the regret rating changed. However, the choice of task changing originates from inverted risk preferences. In Table 5, we calculate the number of subjects’ choice options for change and no change. Moreover, our experimental design (Table 1) was such that option A was a lower-risk choice than option B (higher-risk choice). Meanwhile, we established option B by the computer and then asked participants whether or not they wanted to change from this established choice. Table 5 shows that participants initially chose option B more than option A from trial 1 to trial 4, and they then chose option A more frequently than option B in trial 5 and trial 6, regardless of whether they received left cathodal/right anodal or left anodal/right cathodal stimulation, with the exception of the receipt of left cathodal/right anodal stimulation in trial 3. In these conditions, we found that subjects’ risk preferences changed. In other words, the conversion in risk preferences caused the choices to change, which in turn led to the mean value of regret ratings presenting an inverted U-shaped curve over time.

When people are more adventurous, it is easier to be experience regret based on the choice of a loss outcome in a decision-making context. Therefore, we found that participants altered their choice from no change to change to avoid feelings of regret. The experience of regret will make humans pursue maximizing returns on the one hand and minimizing risks on the other. Bell (1982) and Loomes and Sugden (1982) proposed regret theory, which indicated that humans not only maximized the EU but also minimized regret in their behavioral decisions. Our research further proved regret theory. To reduce the resulting regret, people will seek advantages and avoid disadvantages, which manifests in behavioral decision-making tasks as becoming less risky.

Landman (1987) defined regret as a feeling of a more or less painful cognitive and emotional mental state, which is always sorry for misfortunes, limitations, losses, shortcomings, or mistakes. The research by Purvis et al. (2011) indicated that long-term regret immerses people in painful experiences, reduces people’s subjective well-being and life satisfaction, and harms people’s physical and mental health. Although psychology defines regret as a negative emotion, it has a good influence on humans’ behavioral decision-making, and it makes some of our risky decisions less aggressive, thereby reducing mistakes. Beike et al. (2009) suggested that appropriate regret experiences can improve an individual’s counterfactual thinking ability, which makes us learn from mistakes and better adapt to life. Therefore, regret has a positive impact on investment decision-making, project screening, and plan formulation. Overall, the positive influence of regret on humans’ decision-making cannot be ignored, although this emotion can make us feel depressed and upset. Further study of regret can be carried out along this path to identify the more positive influences of regret in humans’ daily lives.

### Limitations

According to the stimulation effect discussed above, there is more reward than punishment related to the decision-making of participants in our experiment task. Table 1 demonstrates that, even in the loss trial, each choice of obtained outcome is better than the unobtained outcome. Previous research suggested that the left OFC is sensitive to rewards and that the right OFC is sensitive to punishment (O'Doherty et al., 2001; Diekhof et al., 2011; Szatkowska et al., 2011). Thus, our experiment design induced participants’ reward sensitivity, which in turn will generate less regret based on the counterfactual thinking of a known negative outcome *via* modulation of the activity of the subjects’ left OFC. One limitation of our experiment design is that our study only identified the causal relationship between the left OFC and regret. In the future, punishment trials can be performed to aid in the investigation of whether regret has been altered by modulating the activity of the right OFC. In addition, the unilateral stimulation of the OFC to investigate regret requires further study.

## Conclusion

This paper studied the relationship between activity in the OFC and regret in human decision-making by using the gambling tasks designed by Coricelli et al. (2005) and Camille et al. (2010) to induce feelings of regret in subjects and using tDCS to modulate activity in the OFC. The experimental results indicated that there is a significant effect of stimulation type. Furthermore, the regret reported following sham stimulation was higher than that following left anodal/right cathodal stimulation, which indicated that by modulating the OFC activity, there was a reduced intensity of the emotion of regret based on counterfactual thinking. In addition, our study demonstrated that there was an inverted U-shaped curve as the experimental trials continued, which revealed that the intensity of regret changed and was evidenced by the conversion in risk preferences.

## Data Availability Statement

The original contributions presented in the study are included in the article/Supplementary Material; further inquiries can be directed to the corresponding author.

## Ethics Statement

The studies involving human participants were reviewed and approved by Zhejiang University of Finance and Economics ethics committee. The patients/participants provided their written informed consent to participate in this study.

## Author Contributions

PY, XL, HY, and WG designed the experiment and wrote the manuscript. PY, XL, YC, and LZ organized the experiment. PY, YC, HY, and WG analyzed the data. PY, YC, LZ, and WG draw the figures. All authors contributed to the article and approved the submitted version.

## Funding

This work was supported by the Key Projects of Soft Science Research Program of Zhejiang Province (grant no. 2021C25041), the National Natural Science Foundation of China (grant no. 72073117 and 71903169), the Humanities and Social Sciences Foundation of Ministry of Education in China (project no. 18YJC190008), the Zhejiang Provincial Philosophy and Social Science Program (grant no. 19NDQN335YB), and the Key Research of Basic Projects of Zhejiang Provincial Philosophy and Social Science (grant no. 20JDZD021). The experiment was operated in the Center for Economic Behavior and Decision-Making (CEBD), Zhejiang University of Finance and Economics.

## Conflict of Interest

The authors declare that the research was conducted in the absence of any commercial or financial relationships that could be construed as a potential conflict of interest.

## Publisher’s Note

All claims expressed in this article are solely those of the authors and do not necessarily represent those of their affiliated organizations, or those of the publisher, the editors and the reviewers. Any product that may be evaluated in this article, or claim that may be made by its manufacturer, is not guaranteed or endorsed by the publisher.
